# Supplementary material for: Light-triggered switching of liposome surface charge directs delivery of membrane impermeable payloads in vivo
Source: Nat Commun. 2020 Jul 20;11:3638. doi: 10.1038/s41467-020-17360-9 (PMC7371701; doi:10.1038/s41467-020-17360-9)
Supplement: Supplementary file 1 — Supplementary Information [file 41467_2020_17360_MOESM1_ESM.pdf]

SUPPLEMENTARY INFORMATION for

Light-triggered switching of liposome surface charge directs the intracellular delivery of membrane impermeable payloads *in vivo*

Gabriela Arias-Alpizar *et al.*

Supplementary Figures

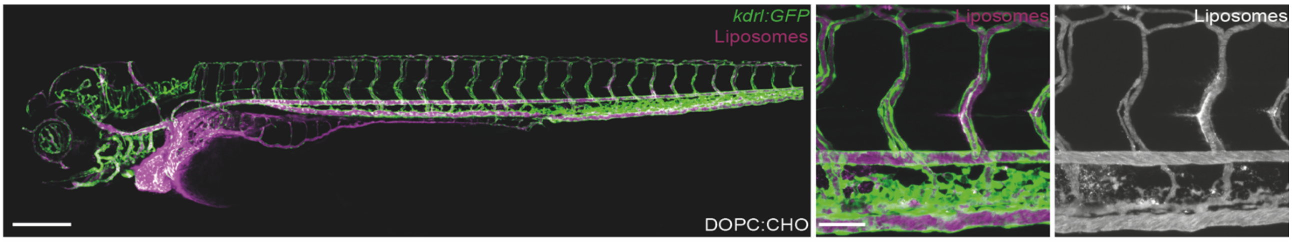

Supplementary Figure 1. Biodistribution of DOPC:cholesterol liposomes. Whole embryo and tissue level views of DOPC:cholesterol (1:1) liposome distribution in *kdrl:GFP* transgenic embryos (2 dpf), 1 hpi. DOPC:cholesterol liposomes freely circulate throughout the vasculature of the embryonic zebrafish and do not appreciably interact with blood-resident macrophages, SECs, or any other endothelial cell types of the embryo. Liposomes contained 1 mol% fluorescent lipid probe, DOPE-LR, for visualization. Scale bars: 200 µm (whole embryo); 50 µm (tissue level).

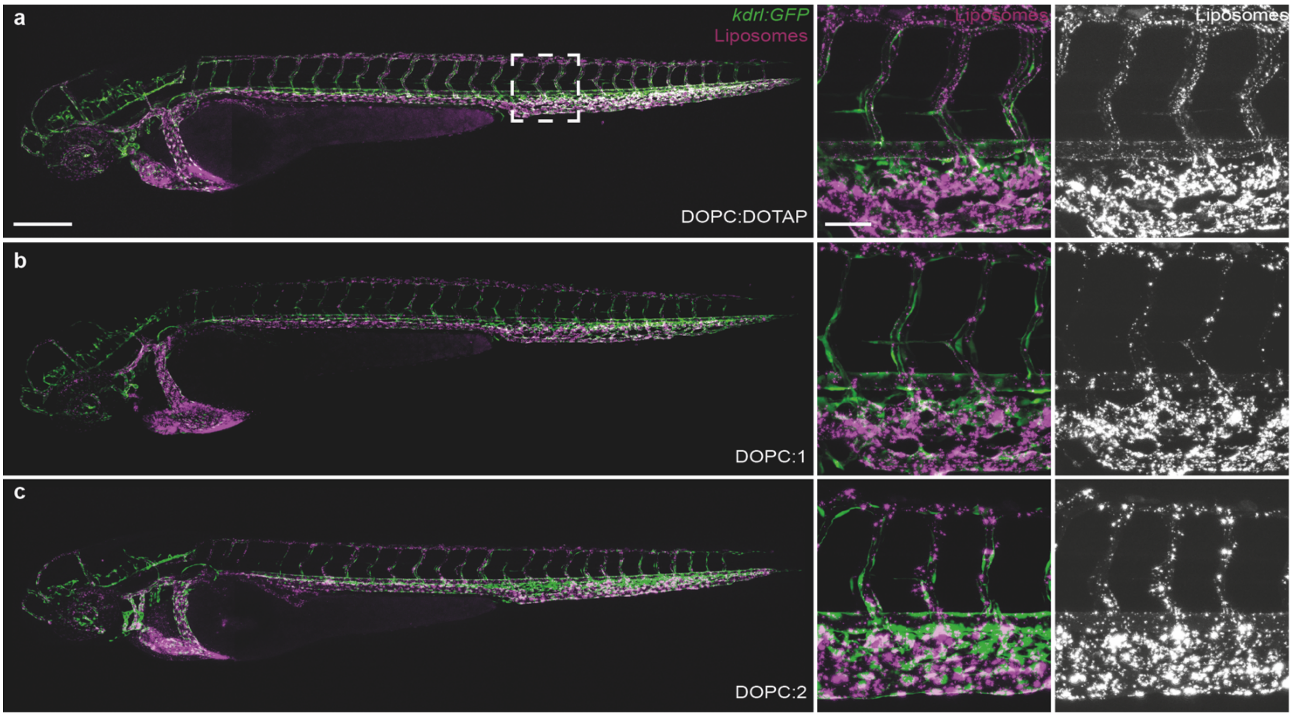

Supplementary Figure 2. Biodistribution of cationic liposomes. Whole embryo and tissue level views of liposome distribution in *kdrl:GFP* transgenic embryos (2 dpf), 1 hpi of **a.** DOPC:DOTAP (1:1), **b.** DOPC:**1** (1:1) and **c.** DOPC:**2** (1:1) liposomes. All three cationic liposome formulations are visible as immobile fluorescent punctae associated with blood vessel walls throughout the vasculature of the zebrafish embryo and are largely removed from circulation. Liposomes contained 1 mol% fluorescent lipid probe, DOPE-LR, for visualization. Scale bars: 200 µm (whole embryo); 50 µm (tissue level).

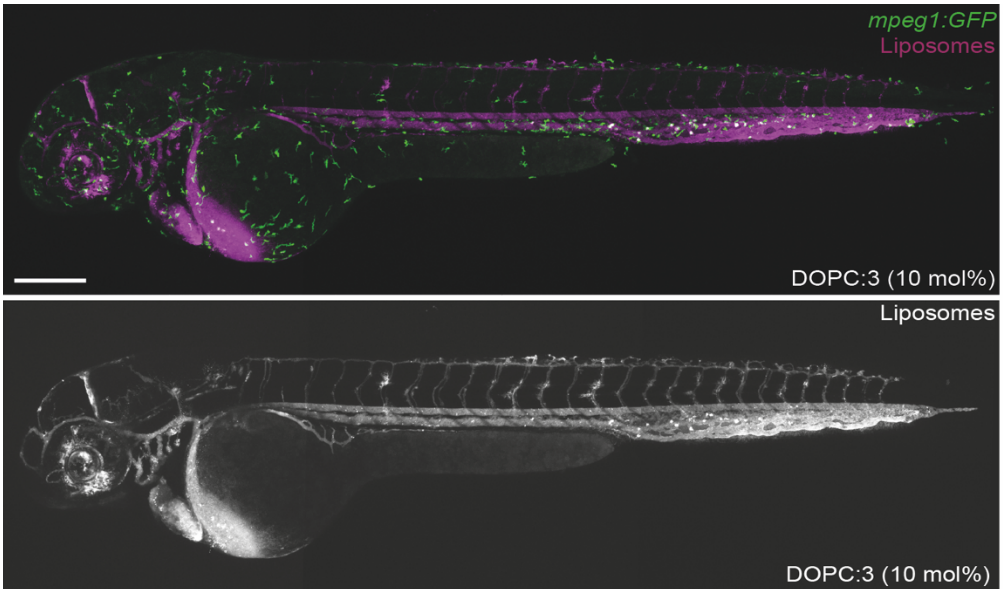

Supplementary Figure 3. Uptake of DOPC:3 (9:1) liposomes within blood resident macrophages. Whole embryo views of DOPC:**3** (9:1) liposome distribution in *mpeg1:GFP* transgenic embryos (2 dpf), 1 hpi. Extensive fluorescence colocalization of liposomes and blood resident macrophages can be observed primarily within the CHT of the embryonic fish. In addition, a significant fraction of liposomes remains in circulation. Liposomes contained 1 mol% fluorescent lipid probe, DOPE-LR, for visualization. Scale bar: 200 µm (whole embryo).

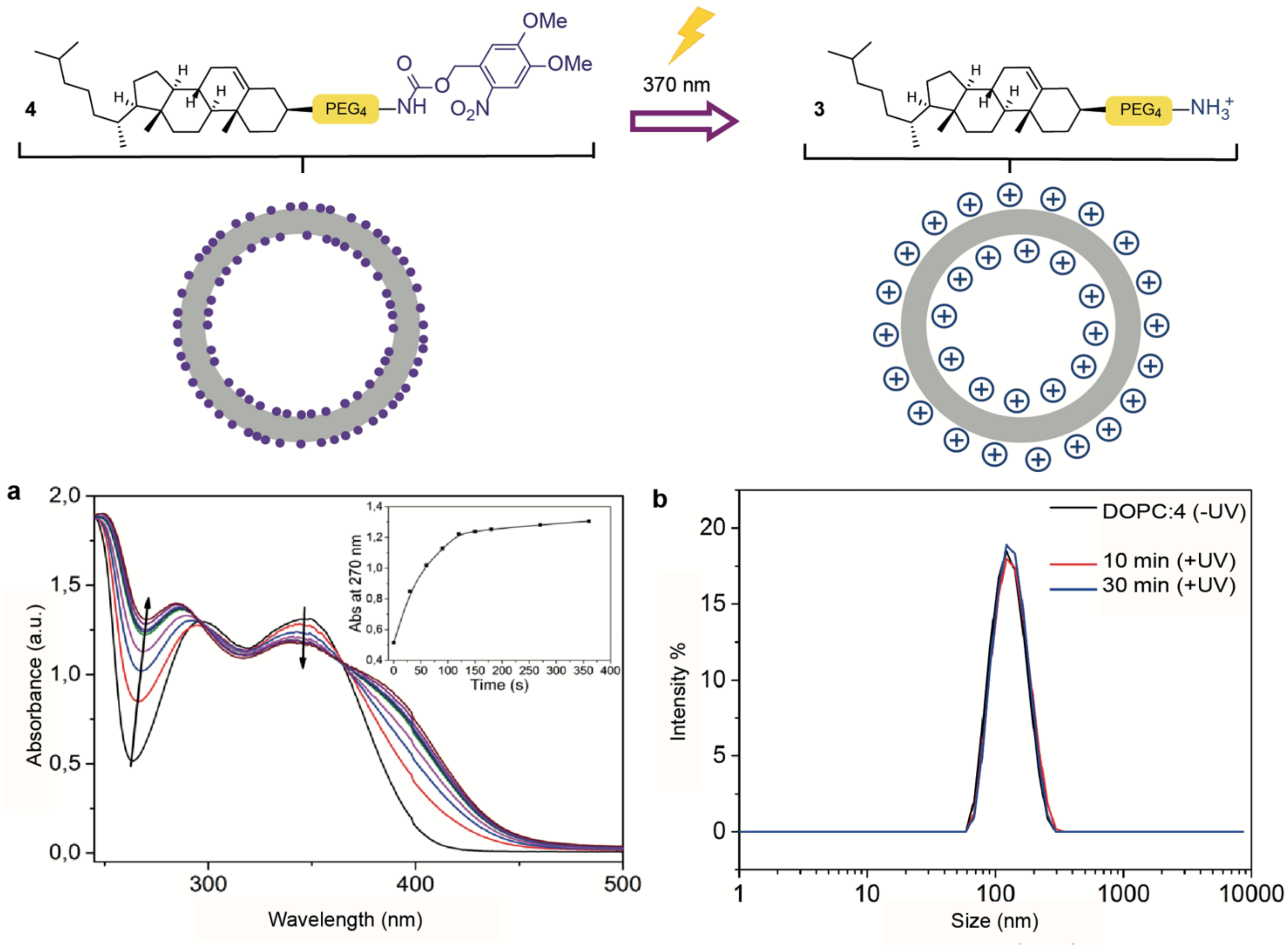

Supplementary Figure 4. Photolysis of 4. a. Time evolution of the UV/Vis spectra of a solution of **4** (200 µM; H_2O/MeCN/^{t}BuOH, 1:1:1) during UV irradiation (370 ± 7 nm, 202 mW cm^{-2}). Inset: Time evolution of the UV absorbance at 270 nm. **b.** DLS spectra of DOPC:**4** (1:1) liposomes before and after light activation (10 and 30 min, 370 ± 7 nm, 202 mW cm^{-2}) and surface charge switching.

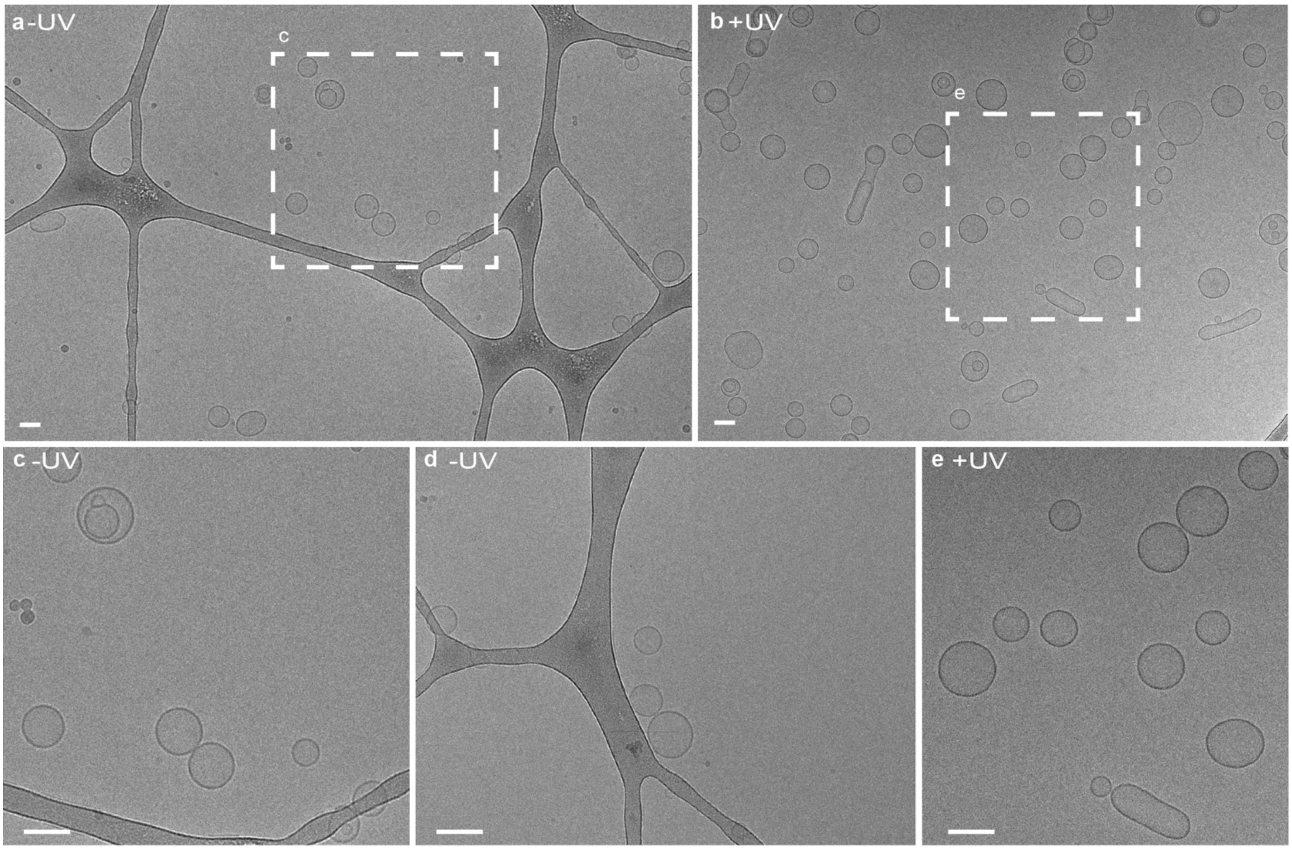

Supplementary Figure 5. Low magnification cryoTEM images of DOPC:4 liposomes before and after UV. CryoTEM images of (empty) DOPC:**4** liposomes (**a,c,d**) before and (**b**,**e**) after *in situ* irradiation (15 min, 370 ± 7 nm, 202 mW cm^{-2}). Note: Two individual cryoTEM panels of the same liposome sample are shown for the -UV sample as observed populations of DOPC:**4** liposomes, prior to UV irradiation, were consistently less than those after UV irradiation despite identical liposome concentrations. Scale bars: 100 nm.

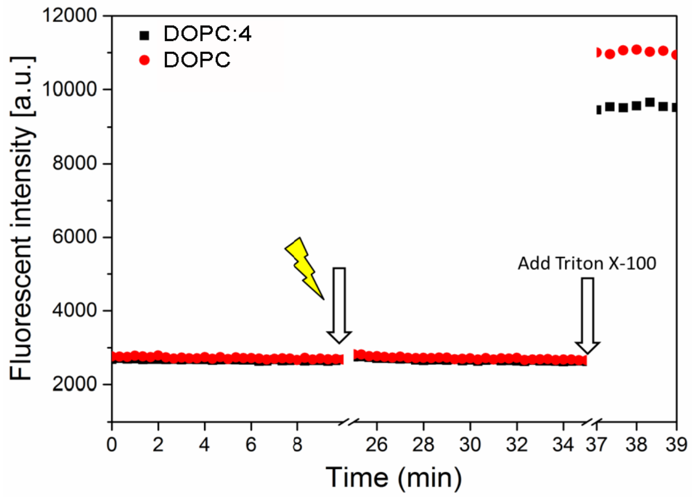

Supplementary Figure 6. Contents (SR-B) leakage. SR-B leakage (and dye de-quenching) from DOPC:**4** liposomes before and after UV irradiation. Fluorescence emission (excitation: 520 nm; emission: 580 nm) was measured every 20 s for 600 s, the sample was then irradiated (20 mins, 370 ± 7 nm, 202 mW cm^{-2}) in a quartz cuvette and fluorescence re-measured for a further 600 s. Triton X-100 (10 µL, 1% w/v) was added to solubilize liposomes and release encapsulated SR-B. DOPC (100% DOPC content) was used as a control to demonstrate contents leakage was not dependent on UV irradiation alone.

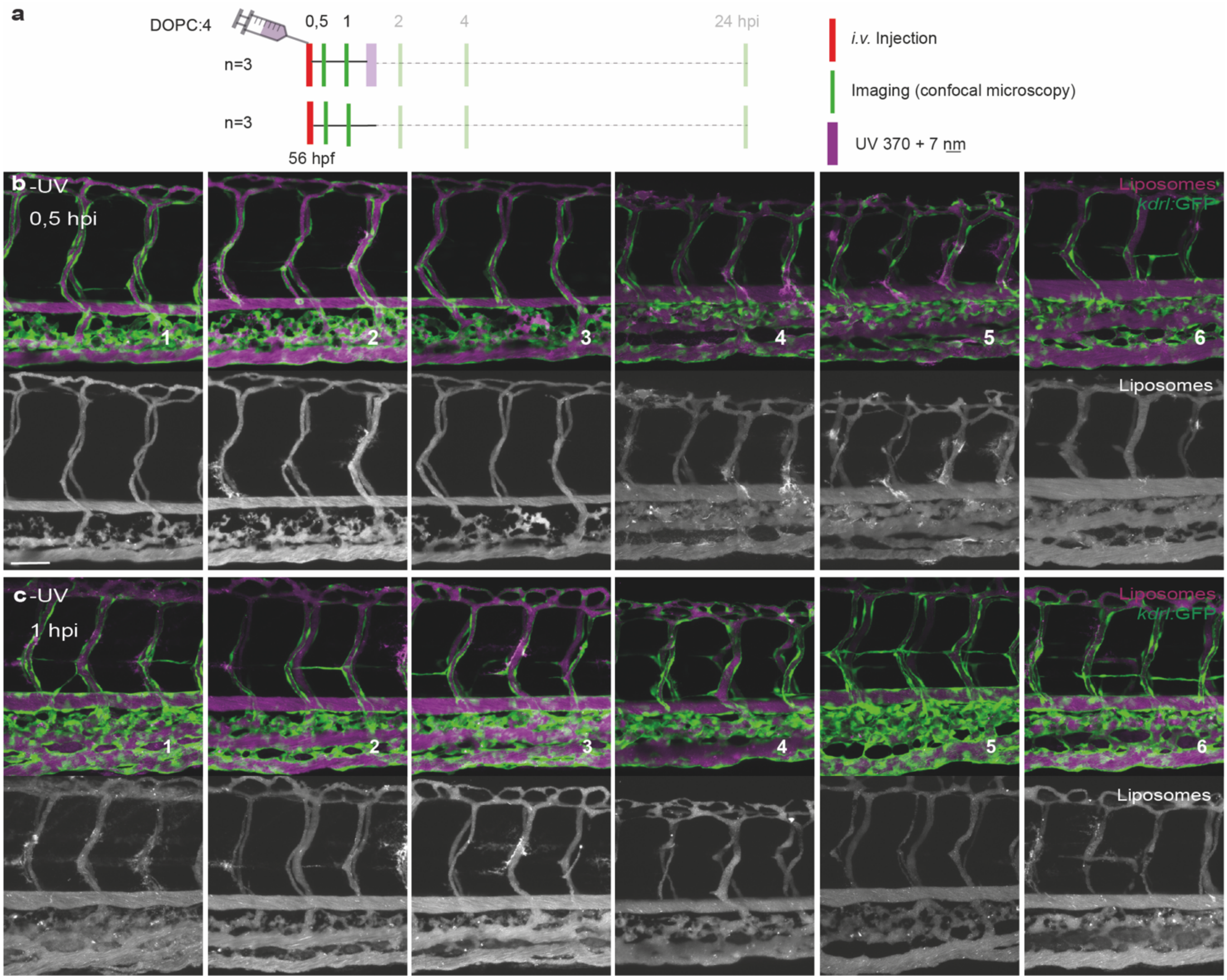

Supplementary Figure 7. Individual tissue level images used for quantification of circulation lifetime decays. **a.** Time schedule of injections, time course imaging and UV irradiation used to quantify liposome circulation lifetime decay. Tissue level views of DOPC:**4** liposome distribution in *kdrl:GFP* transgenic zebrafish embryos at **b.** 0.5 hpi (n=6), **c.** 1 hpi (n=6). Quantification of circulation lifetime decays performed as previously described.[8] Liposomes contained 1 mol% fluorescent lipid probe, DOPE-LR, for visualization. Scale bar: 50 µm (tissue level).

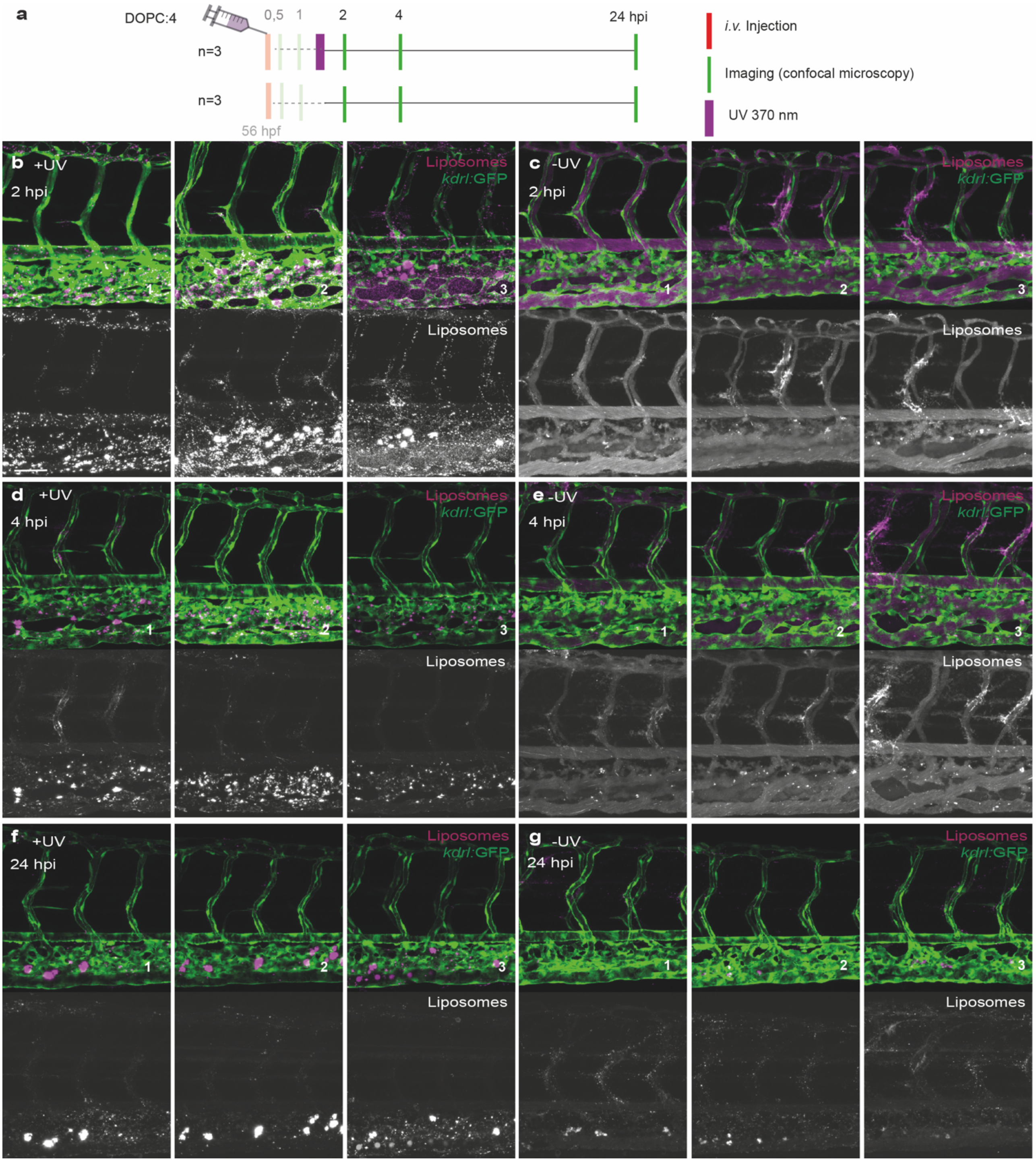

Supplementary Figure 8. Individual tissue level images used for quantification of circulation lifetime decays. **a.** Time schedule of injections, time course imaging and UV irradiation used to quantify liposome circulation lifetime decay. Tissue level views of DOPC:**4** liposome distribution in *kdrl:GFP* transgenic zebrafish embryos at **c.** 2 hpi (n=3), e. 4 hpi (n=3), **g.** 24 hpi (n=3) in the absence of UV irradiation, and of DOPC:**4**→**3** liposomes at **b.** 2 hpi (n=3), **d.** 4 hpi (n=3), **f.** 24 hpi (n=3) following UV irradiation at 1.1 hpi. Quantification of circulation lifetime decays performed as previously described.[8] Liposomes contained 1 mol% fluorescent lipid probe, DOPE-LR, for visualization. Scale bar: 50 µm (tissue level).

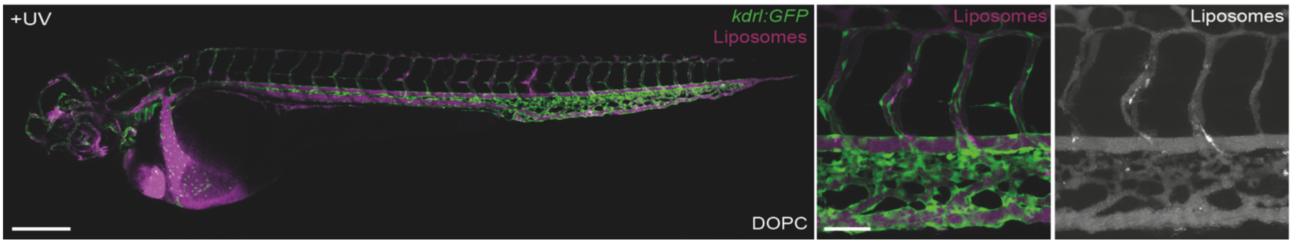

Supplementary Figure 9. Biodistribution of freely circulating DOPC liposomes following UV irradiation. Whole embryo and tissue level views of DOPC (100% DOPC content) liposome distribution in *kdrl:GFP* transgenic embryos (2 dpf), 1 hpi. following UV *in situ* irradiation (15 min, 370 ± 7 nm, ~90 mW cm^{-2}, ~2.4 J per embryo). The biodistribution of DOPC liposomes was unaffected by UV irradiation and liposomes remained in circulation. Liposomes contained 1 mol% fluorescent lipid probe, DOPE-LR, for visualization. Scale bars: 200 µm (whole embryo); 50 µm (tissue level).

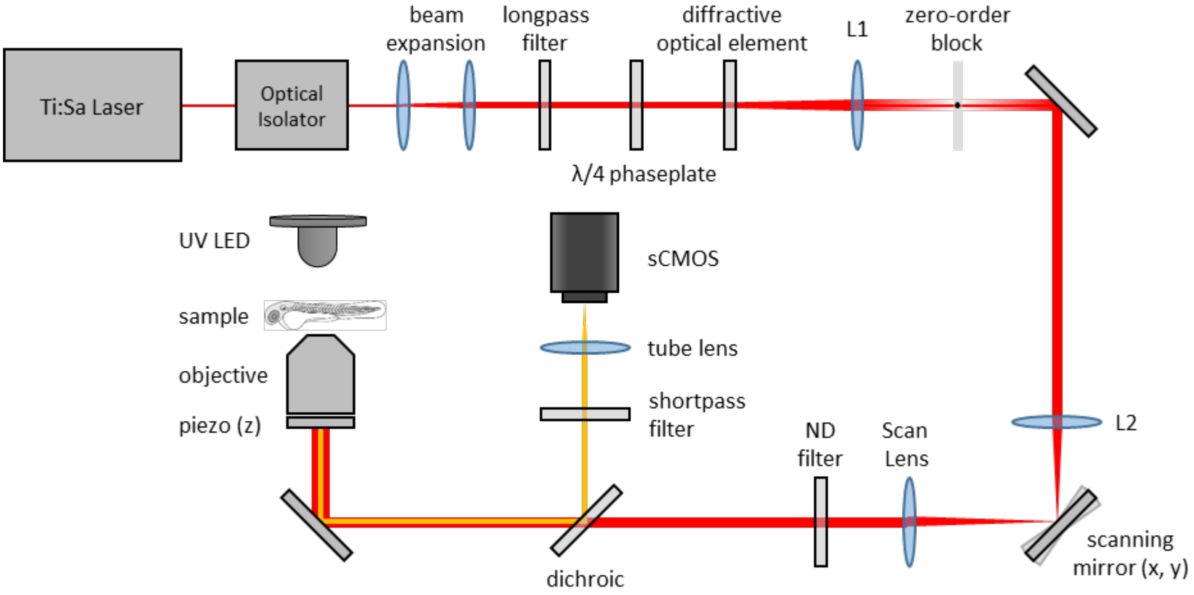

Supplementary Figure 10. Schematic of the two-photon multifocal microscope setup for real-time imaging during UV illumination. The laser beam was diffracted by a diffractive optical element (DOE, custom-made, Holo-eye) in an array of 25 x 25 individual foci. The DOE image was collected by a lens (L1) and the zero-order diffraction was blocked by a droplet of tin. The first diffraction order is focused (L2) behind the scanning mirror (FSM-300, Newport), driven by an Archimedean spiral function. A scan lens (f=200 mm, Newport) collected the reflected light from the scanning mirror and the light was focused in the sample by a 25X water-dipping objective (Apochromat 25XC W, Nikon). The objective was positioned onto a piezo stage (P-726 PIFOC, PI) for z-stack measurements. Excitation power was modulated by neutral density filters (Thorlabs). The UV LED was positioned approximately 1.5 cm above the sample and on/off-times are timed by the same data acquisition card (USB-6226, National Instruments) that triggered the 4.2 megapixel sCMOS camera (PRIME BSI, Photometrics). Emission light collected by the objective was separated from the excitation path by a dichroic mirror (700 dcxr, Chroma). After passing a shortpass emission filter (FF01-527/70-25, Semrock), the image was focused by a tube lens (f=200 mm, Newport) onto the camera. During one camera exposure (50 ms), the excitation plane (250 x 250 µm) was completely illuminated by the foci.

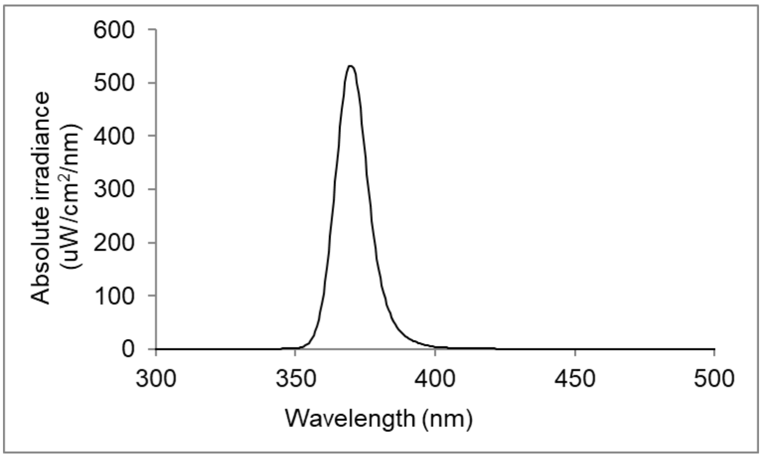

Supplementary Figure 11. UV-Vis spectrum of the H2A1-H375-S LED (Roithner Lasertechnik, Austria). The optical power density of the LED light source was determined using an integrating sphere setup. For this, the LED was positioned precisely 5 cm above the 6.0 mm aperture of an integrating sphere. The sphere was connected by an optical to a UV-Vis spectrometer. The setup was calibrated using a NIST-traceable calibration light source.

Supplementary Tables

Composition	mol%	Buffer	Size		Zeta potential (mV)
			D_h (nm)	PDI	
DOPC:1	95:5	water	116.9	0.108	-7.70
	90:10	water	117.7	0.082	-1.05
	75:25	water	109.5	0.139	24.40
	50:50	water	119.7	0.068	30.13
DOPC:2	95:5	water	113.4	0.098	-9.25
	90:10	water	114.4	0.085	-4.15
	75:25	water	117.8	0.100	13.84
	50:50	water	117.4	0.101	38.10
DOPC:3	95:5	water	112.5	0.058	-8.25
	90:10	water	102.8	0.074	-4.94
	75:25	water	110.8	0.134	15.07
	50:50	water	122.7	0.156	48.53
DOPC:DOTAP	50:50	water	123.9	0.050	44.83
**DOPC:4 (before UV)**	50:50	HEPES	105.2	0.085	-8.07
**DOPC:4 (after UV)**	50:50	HEPES	115.2	0.101	25.93

Supplementary Table 1. Composition, size and measured zeta potentials of liposomes. All reported DLS measurements and zeta potentials are the average of three measurements. For measurements monitoring changes following light activation, liposomes were irradiated (20 mins, 370 nm ± 7 nm, 202 mW cm^{-2}) in quartz cuvettes, with the LED mounted at a distance of 1 cm from the sample. Reported sizes and zeta potentials are for individual liposome samples but representative over multiple sample preparations. The number of individual liposome preparations varied depending on liposome formulation. In the case of DOPC:**4** liposomes (prior to UV irradiation), sizes and zeta potentials did not significantly deviate across >5 independent formulations. This included variations in DOPC supplier, $CHCl_3$ lipid stock solutions and synthesized batches of **4**. Batch-to-batch variation in the measured zeta potential of DOPC:**4**→**3** liposomes (*i.e.* after UV irradiation) resulted in a liposome surface charges ranging from +20 to +35 mV ζ-potential. The same liposome sample was measured before and after UV irradiation.

Supplementary Methods

Chemical synthesis of 1-4.

Cholesteryl Fmoc-glycinate[11]

Cholesterol (500 mg, 1.29 mmol), Fmoc-Gly-OH (577 mg, 1.94 mmol, 1.5 eq.) and EDC•HCl (744 mg, 3.88 mmol, 3.0 eq.) were dissolved in dry CH_2Cl_2 (25 mL). A catalytic amount of DMAP (16 mg, 129 µmol, 0.1 eq.) was added and the reaction mixture was stirred for 24 h at room temperature. CH_2Cl_2 (25 mL) was added and the solution was washed with 1 M HCl (2 x 50 mL), water (50 mL) and brine (50 mL). The organic phase was dried over sodium sulfate and solvent removed under reduced pressure. The crude mixture was purified by flash-column chromatography to obtain cholesteryl Fmoc-glycinate (604 mg, 0.91 mmol, 70%) as a colorless solid.

TLC (Petroleum ether (40 – 60 °C)/ethyl acetate = 4:1): R_f = 0.34.
^{1}H NMR (400 MHz, $CDCl_3$): δ (ppm) = 7.77 (d, J = 7.5 Hz, 2H, H_{Ar}), 7.61 (d, J = 7.5 Hz, 2H, H_{Ar}), 7.40 (t, J = 7.5 Hz, 2H, H_{Ar}), 7.32 (td, J = 7.5, 1.0 Hz, 2H, H_{Ar}), 5.38 (d, J = 4.0 Hz, 1H, C=CH), 5.29 (t, J = 5.4 Hz, 1H, NH), 4.77 – 4.63 (m, 1H, OCH_{chol}), 4.40 (d, J = 7.1 Hz, 2H, $CH_{2,\,Fmoc}$), 4.24 (t, J = 7.1 Hz, 1H, CH_{Fmoc}), 3.98 (d, J = 5.4 Hz, 2H, $C\underline{H}_2NH$), 2.34 (d, J = 7.8 Hz, 2H, $C\underline{H}_2C{=}CH$), 2.09 – 1.04 (m, 26H, H_{chol}), 1.02 (s, 3H, $C\underline{H}_3CC{=}CH$), 0.91 (d, J = 6.5 Hz, 3H, $C\underline{H}_3CHCH$), 0.87 (d, J = 1.8 Hz, 3H, $C\underline{H}_3CHCH_3$), 0.86 (d, J = 1.8 Hz, 3H, $CH_3CHC\underline{H}_3$), 0.68 (s, 3H, $C\underline{H}_3CCH$).

Cholesteryl glycinate, 1[11]

Cholesteryl Fmoc-glycinate (201 mg, 302 µmol) was dissolved in CH_2Cl_2/diethylamine (1:1, 4 mL) and stirred for 3 h at room temperature. The solvent was removed under reduced pressure and remnant diethylamine further co-evaporated with MeOH (3 x 10 mL). The crude product was dissolved in CH_2Cl_2 and purified by flash-column chromatography (CH_2Cl_2/methanol 100:0 → 99:1 → 95:5) to obtain **1** (87.4 mg, 197 µmol, 65%) as a pale yellow solid.

TLC (CH_2Cl_2/methanol = 9:1): R_f = 0.34.
^{1}H NMR (400 MHz, $CDCl_3$): δ (ppm) = 5.38 (d, J = 3.7 Hz, 1H, C=CH), 4.73 – 4.60 (m, 1H, OCH_{chol}), 3.41 (s, 2H, $C\underline{H}_2NH_2$), 2.32 (d, J = 7.9 Hz, 2H, $C\underline{H}_2C{=}CH$), 2.06 – 1.04 (m, 26H, H_{chol}), 1.01 (s, 3H, $C\underline{H}_3CC{=}CH$), 0.91 (d, J = 6.3 Hz, 3H, $C\underline{H}_3CHCH$), 0.87 (s, 3H, $C\underline{H}_3CHCH_3$), 0.85 (s, 3H, $CH_3CHC\underline{H}_3$), 0.67 (s, 3H, $C\underline{H}_3CCH$).

Cholesteryl-PEG$_2$-NHFmoc

Cholesterol (100 mg, 259 µmol), [2-(2-(Fmoc-amino)ethoxy)ethoxy]acetic acid (Fmoc-PEG$_2$-COOH, 100 mg, 259 µmol) and DMAP (6.00 mg, 49.1 µmol, 0.2 eq.) were dissolved in dry CH_2Cl_2 (2 mL). A solution of EDC•HCl (99.2 mg, 517 µmol, 2 eq.) and DIPEA (0.07 mL, 389 µmol, 1.5 eq.) in dry CH_2Cl_2 (4 mL) was added dropwise to the reaction mixture at 0 °C. The solution was allowed to warm up to room temperature and stirred for a further 18 h. The reaction mixture was diluted with CH_2Cl_2 (20 mL), washed with 1 M aq. HCl (2 x 30 mL), water (30 mL) and brine (30 mL). The organic phase was dried over magnesium sulfate and the solvent removed under reduced pressure. The crude product was purified by flash-column chromatography (petroleum ether (40 – 60 °C)/EtOAc; 1:0 → 4:1 → 2:1 → 1:1) to obtain cholesterol-PEG$_2$-NHFmoc (125 mg, 166 µmol, 64%) as a colorless solid.

TLC (CH_2Cl_2/methanol = 17:3): R_f = 0.21.
^{1}H NMR (400 MHz, $CDCl_3$): δ (ppm) = 7.76 (d, J = 7.5 Hz, 2H, H_{Ar}), 7.62 (d, J = 7.4 Hz, 2H, H_{Ar}), 7.39 (t, J = 7.5 Hz, 2H, H_{Ar}), 7.31 (td, J = 7.4, 0.9 Hz, 2H, H_{Ar}), 5.52 (t, J = 5.2 Hz, 1H, NH), 5.32 (d, J = 4.2 Hz, 1H, C=CH), 4.77 – 4.66 (m, 1H, OCH_{chol}), 4.37 (d, J = 7.2 Hz, 2H, $CH_{2,\,Fmoc}$), 4.22 (t, J = 7.2 Hz, 1H, CH_{Fmoc}),

4.11 (s, 2H, CH_2COO), 3.78 – 3.64 (m, 4H, OCH_2CH_2O), 3.60 (t, J = 5.1 Hz, 2H, $NHCH_2C\underline{H}_2$), 3.43 (q, J = 5.1 Hz, 2H, $NHC\underline{H}_2$), 2.32 (d, J = 7.9 Hz, 2H, $C\underline{H}_2C{=}CH$), 2.10 – 1.00 (m, 26H, H_{chol}), 0.98 (s, 3H, $C\underline{H}_3CC{=}CH$), 0.91 (d, J = 6.5 Hz, 3H, $C\underline{H}_3CHCH$), 0.88 (d, J = 1.9 Hz, 3H, $C\underline{H}_3CHCH_3$), 0.86 (d, J = 1.8 Hz, 3H, $CH_3CHC\underline{H}_3$), 0.66 (s, 3H, $C\underline{H}_3CCH$).
^{13}C NMR (101 MHz, $CDCl_3$): δ (ppm) = 170.0, 156.6, 144.1, 141.4, 139.3, 127.7, 127.1, 125.2, 123.0, 120.0, 91.0, 74.8, 71.0, 70.4, 70.3, 68.8, 66.8, 56.7, 56.2, 50.0, 47.3, 42.3, 41.0, 39.7, 39.6, 38.1, 36.9, 36.5, 36.2, 35.9, 31.9, 31.8, 28.3, 28.1, 27.8, 24.3, 23.9, 22.9, 22.6, 21.1, 19.3, 18.8, 11.9.
HR-MS (ESI^+): calc. ($C_{48}H_{67}NO_6Na$): m/z = 776.48606, found: m/z = 776.48630.

Cholesteryl-PEG$_2$-NH$_2$, 2
Cholesterol-PEG$_2$-NHFmoc (104 mg, 138 µmol) was dissolved in CH_2Cl_2/diethylamine (1:1, 4 mL) and stirred for 3 h at room temperature. The solvent was removed under reduced pressure and diethylamine residues co-evaporated with methanol (3 x 30 mL). The crude was dissolved in CH_2Cl_2 and purified by flash-column chromatography (CH_2Cl_2/methanol 1:0 → 9:1 → 17:3) to obtain **2** (28.4 mg, 53.4 µmol, 39%) as a pale yellow solid.

TLC (CH_2Cl_2/methanol = 17:3): R_f = 0.21.
^{1}H NMR (400 MHz, $CDCl_3$): δ (ppm) = 5.37 (s, 1H, $C{=}CH$), 4.77 – 4.53 (m, 1H, OCH_{chol}), 4.12 (s, 2H, CH_2COO), 3.85 (s, 2H, $NH_2CH_2C\underline{H}_2$), 3.72 (s, 4H, OCH_2CH_2O), 3.26 (s, 2H, $NH_2C\underline{H}_2$), 2.31 (d, J = 7.6 Hz, 2H, $C\underline{H}_2C{=}CH$), 2.10 – 1.03 (m, 26H, H_{chol}), 1.00 (s, 3H, $C\underline{H}_3CC{=}CH$), 0.90 (d, J = 6.0 Hz, 3H, $C\underline{H}_3CHCH$), 0.86 (s, 3H, $C\underline{H}_3CHCH_3$), 0.84 (s, 3H, $CH_3CHC\underline{H}_3$), 0.66 (s, 3H, $C\underline{H}_3CCH$).

Cholesteryl-PEG$_4$-N$_3$
Cholesterol (194 mg, 502 µmol), 14-azido-3,6,9,12-tetraoxatetradecanoic acid (139 mg, 502 µmol) and a catalytic amount of DMAP (6 mg, 50 µmol, 0.10 eq.) were dissolved in dry CH_2Cl_2 (5 mL). A solution of EDC•HCl (192 mg, 1.00 mmol, 2 eq.) and DIPEA (0.13 mL, 753 µmol, 1.5 eq.) in dry CH_2Cl_2 (5 mL) was added to the reaction mixture dropwise at 0 °C. The solution was stirred for 20 h at room temperature. CH_2Cl_2 (40 mL) was added and the solution was washed with 1 M aq. HCl (2 x 50 mL) and brine (50 mL). The organic phase was dried over magnesium sulfate and the solvent was removed under reduced pressure. The crude was purified by flash-column chromatography (petroleum ether (40 – 60 °C)/ethyl acetate 1:0 → 4:1 → 3:1 → 2:1) to obtain cholesterol-PEG$_4$-N$_3$ (127 mg, 197 µmol, 39%) as a colorless solid.

TLC (petroleum ether (40 – 60 °C)/ethyl acetate = 1:1): R_f = 0.37.
^{1}H NMR (400 MHz, $CDCl_3$): δ (ppm) = 5.37 (s, 1H, $C{=}CH$), 4.80 – 4.60 (m, 1H, OCH_{chol}), 4.12 (s, 2H, CH_2COO), 3.78 – 3.60 (m, 14H, OCH_2), 3.39 (d, J = 2.2 Hz, 2H, CH_2N_3), 2.33 (d, J = 7.4 Hz, 2H, $C\underline{H}_2C{=}CH$), 2.07 – 0.93 (m, 26H, H_{chol}), 1.01 (s, 3H, $C\underline{H}_3CC{=}CH$), 0.91 (s, 3H, $C\underline{H}_3CHCH$), 0.86 (s, 3H, $C\underline{H}_3CHCH_3$), 0.86 (s, 3H, $CH_3CHC\underline{H}_3$), 0.67 (s, 3H, $C\underline{H}_3CCH$).
^{13}C NMR (75 MHz, $CDCl_3$): δ (ppm) = 169.9, 139.4, 122.9, 74.6, 70.8, 70.7, 70.7, 70.6, 70.0, 68.8, 56.7, 56.1, 50.7, 50.0, 42.3, 39.7, 39.5, 38.1, 36.9, 36.6, 36.2, 35.8, 31.9, 31.8, 28.2, 28.0, 27.7, 24.3, 23.8, 22.8, 22.6, 21.0, 19.3, 18.7, 11.9.
HR-MS (ESI^+): calc. ($C_{37}H_{63}N_3O_6Na$): m/z = 668.46091, found: m/z = 668.46063.

Cholesteryl-PEG$_4$-NH$_2$, 3
Cholesterol-PEG$_4$-N$_3$ (105 mg, 163 µmol) was dissolved in dry THF (8 mL) and a solution of 1 M trimethylphosphine in toluene (0.49 mL, 489 µmol, 3 eq.) added dropwise at 0 °C. The solution was warmed to room temperature and stirred for 3.5 h. 1 M aq. NaOH (25 mL) was then added and the mixture stirred for 1 h at room temperature. The crude product was extracted into CH_2Cl_2 (3 x 30 mL) and the organic phase washed with brine (30 mL) and dried over magnesium sulfate. The solvent was removed under reduced pressure. The crude product was purified by flash-column chromatography (CH_2Cl_2/MeOH

1:0 → 99:1 → 97:3 → 95:5 → 9:1, eluent contained 1% of a 33% aqueous ammonia solution) to obtain **3** (37.4 mg, 60.2 µmol, 37%) as a colorless solid.

TLC (CH_2Cl_2/methanol/33% aq. NH_3 = 9:1:0.1): R_f = 0.21.
^{1}H NMR (400 MHz, $CDCl_3$): δ (ppm) = 5.36 (d, J = 3.9 Hz, 1H, C=CH), 4.75 – 4.60 (m, 1H, OCH_{chol}), 4.10 (s, 2H, CH_2COO), 3.77 – 3.56 (m, 1H, OCH_2CH_2O), 3.50 (t, J = 5.2 Hz, 1H, $C\underline{H}_2CH_2NH_2$), 2.85 (t, J = 5.2 Hz, 1H, $CH_2C\underline{H}_2NH_2$), 2.31 (d, J = 7.8 Hz, 2H, $C\underline{H}_2C{=}CH$), 2.07 – 1.01 (m, 26H, H_{chol}), 0.99 (s, 3H, $C\underline{H}_3CC{=}CH$), 0.89 (d, J = 6.5 Hz, 3H, $C\underline{H}_3CHCH$), 0.85 (d, J = 1.8 Hz, 3H, $C\underline{H}_3CHCH_3$), 0.83 (d, J = 1.8 Hz, 3H, $CH_3CHC\underline{H}_3$), 0.65 (s, 3H, $C\underline{H}_3CCH$).
^{13}C NMR (75 MHz, MeOD-d_4): δ (ppm) = 171.9, 141.8, 140.2, 123.8, 122.2, 76.3, 72.1, 71.4, 71.2, 70.9, 70.8, 70.4, 69.2, 68.9, 67.5, 57.8, 57.2, 52.5, 51.3, 51.2, 43.2, 42.7, 40.8, 40.4, 40.2, 38.8, 38.3, 37.4, 37.1, 36.8, 32.9, 32.8, 32.0, 30.5, 29.1, 28.9, 28.5, 25.1, 24.7, 23.2, 22.9, 21.9, 19.8, 19.2, 12.3.
HR-MS (ESI^+): calc. ($C_{37}H_{66}NO_6$): m/z = 620.48847, found: m/z = 620.48854.

Cholesteryl-PEG$_4$-NVOC, 4
Cholesterol-PEG$_4$-NH_2 (39.5 mg, 60.2 µmol) and DIPEA (16 µL, 90.3 µmol, 1.5 eq.) were dissolved in CH_2Cl_2 (3 mL). A solution of 4,5-dimethoxy-2-nitrobenzyl chloroformate (33.2 mg, 120 µmol, 2 eq.) in CH_2Cl_2 (3 mL) was added dropwise at 0 °C and the reaction mixture allowed to warm to room temperature and stirred for a further 18 h. CH_2Cl_2 (10 mL) was added and the solution was washed with a 1 M aq. HCl (10 mL) and brine (10 mL). The organic phase was dried over magnesium sulfate and the solvent removed under reduced pressure. The crude was purified by flash-column chromatography (petroleum ether (40 – 60 °C)/ethyl acetate 1:0 → 3:1 → 1:1 → 1:3 → 0:1) to obtain **4** (10.6 mg, 12.3 µmol, 21%) as a colorless solid.

TLC (ethyl acetate): R_f = 0.42.
^{1}H NMR (300 MHz, $CDCl_3$): δ (ppm) = 7.71 (s, 1H, H_{Ar}), 7.04 (s, 1H, H_{Ar}), 5.67 (s, 1H, NH), 5.52 (s, 2H, $CH_{2,NVOC}$), 5.37 (d, J = 3.9 Hz, 1H, C=CH), 4.77 – 4.58 (m, 1H, OCH_{chol}), 4.10 (s, 2H, CH_2COO), 3.99 (s, 3H, OMe), 3.95 (s, 3H, OMe), 3.76 – 3.62 (m, 12H, OCH_2CH_2O), 3.59 (d, J = 4.9 Hz, 2H, $NHCH_2C\underline{H}_2$), 3.47 – 3.37 (m, 2H, $NHC\underline{H}_2$), 2.32 (d, J = 7.8 Hz, 2H, $C\underline{H}_2C{=}CH$), 2.10 – 1.03 (m, 26H, H_{chol}), 1.01 (s, 3H, $C\underline{H}_3CC{=}CH$), 0.91 (d, J = 6.4 Hz, 3H, $C\underline{H}_3CHCH$), 0.88 (d, J = 1.1 Hz, 3H, $C\underline{H}_3CHCH_3$), 0.85 (d, J = 1.0 Hz, 3H, $CH_3CHC\underline{H}_3$), 0.67 (s, 3H, $C\underline{H}_3CCH$).
^{13}C NMR (75 MHz, $CDCl_3$): δ (ppm) = 170.0, 156.1, 153.7, 148.1, 139.8, 139.5, 128.6, 123.1, 110.2, 108.3, 74.8, 71.0, 70.7, 70.4, 70.2, 68.9, 63.6, 56.8, 56.7, 56.5, 56.2, 50.1, 42.4, 41.1, 39.8, 39.6, 38.2, 37.0, 36.7, 36.3, 35.9, 32.01, 31.95, 28.4, 28.1, 27.9, 24.4, 24.0, 23.0, 22.7, 21.1, 19.4, 18.8, 12.0.
HR-MS (ESI^+): calc. ($C_{47}H_{74}N_2O_{12}Na$): m/z = 881.51340, found: m/z = 881.51353.

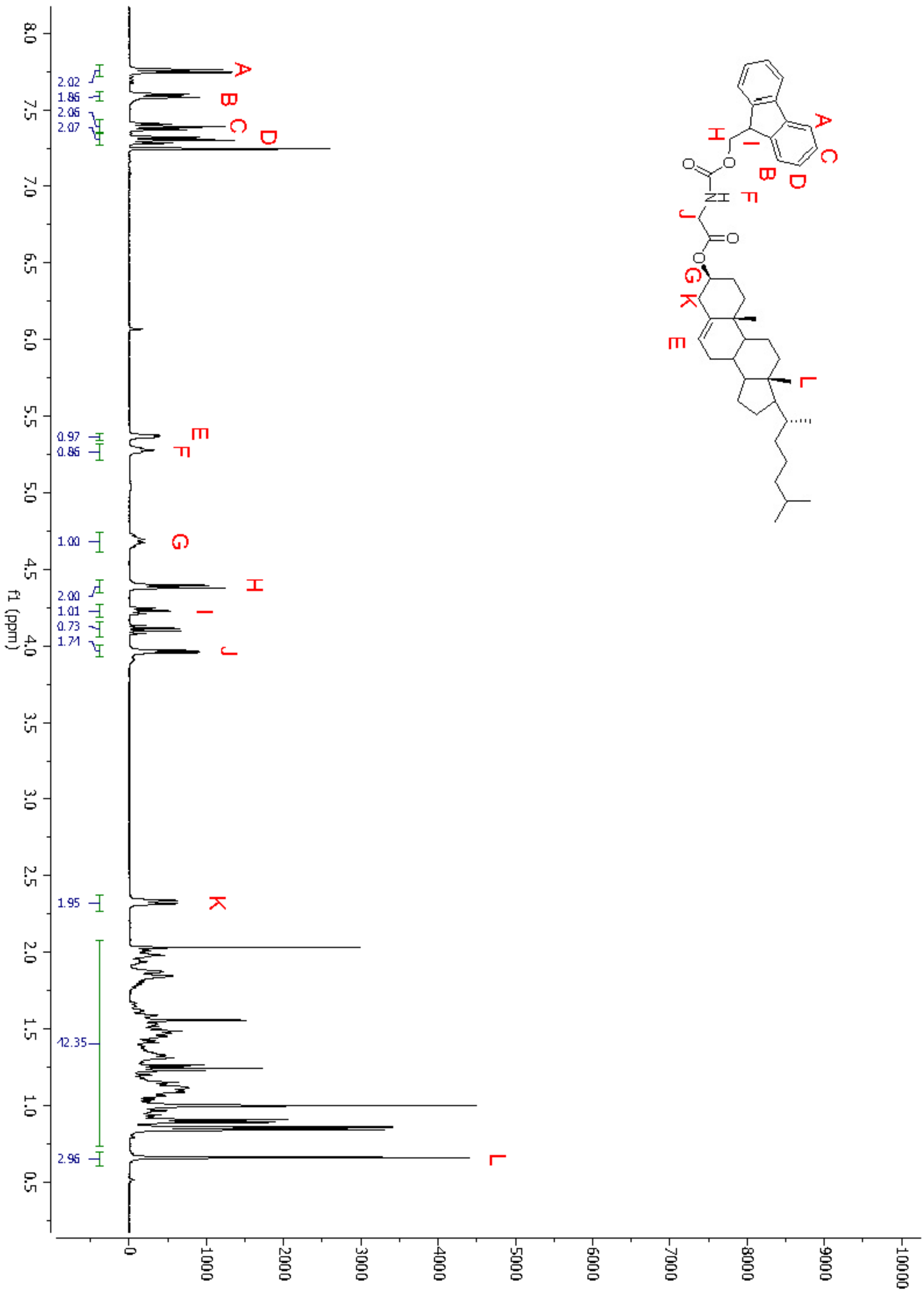

Supplementary Figure 12. ^{1}H-NMR of Cholesteryl Fmoc-glycinate

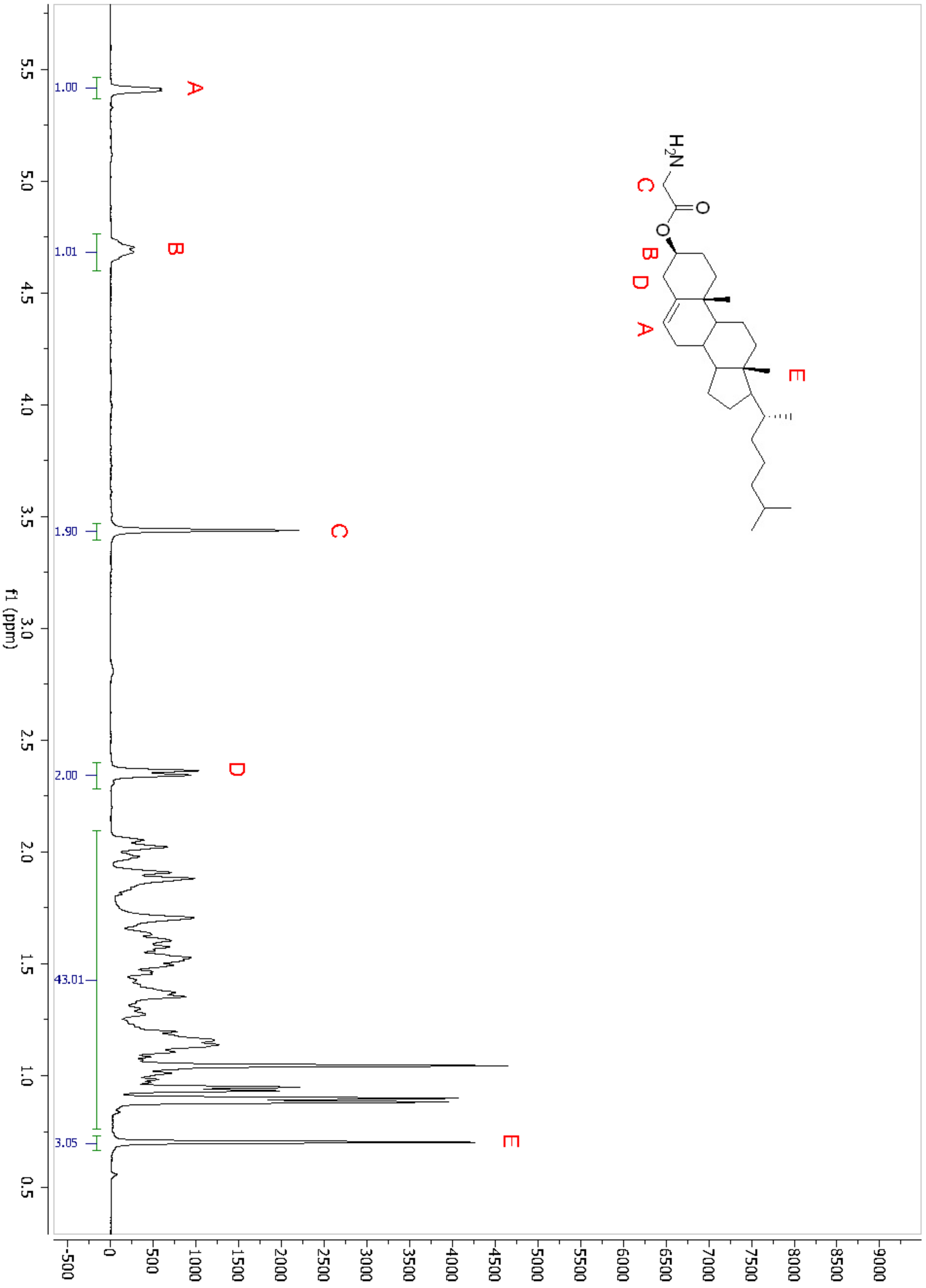

Supplementary Figure 13. ^{1}H-NMR of Cholesteryl glycinate, **1**

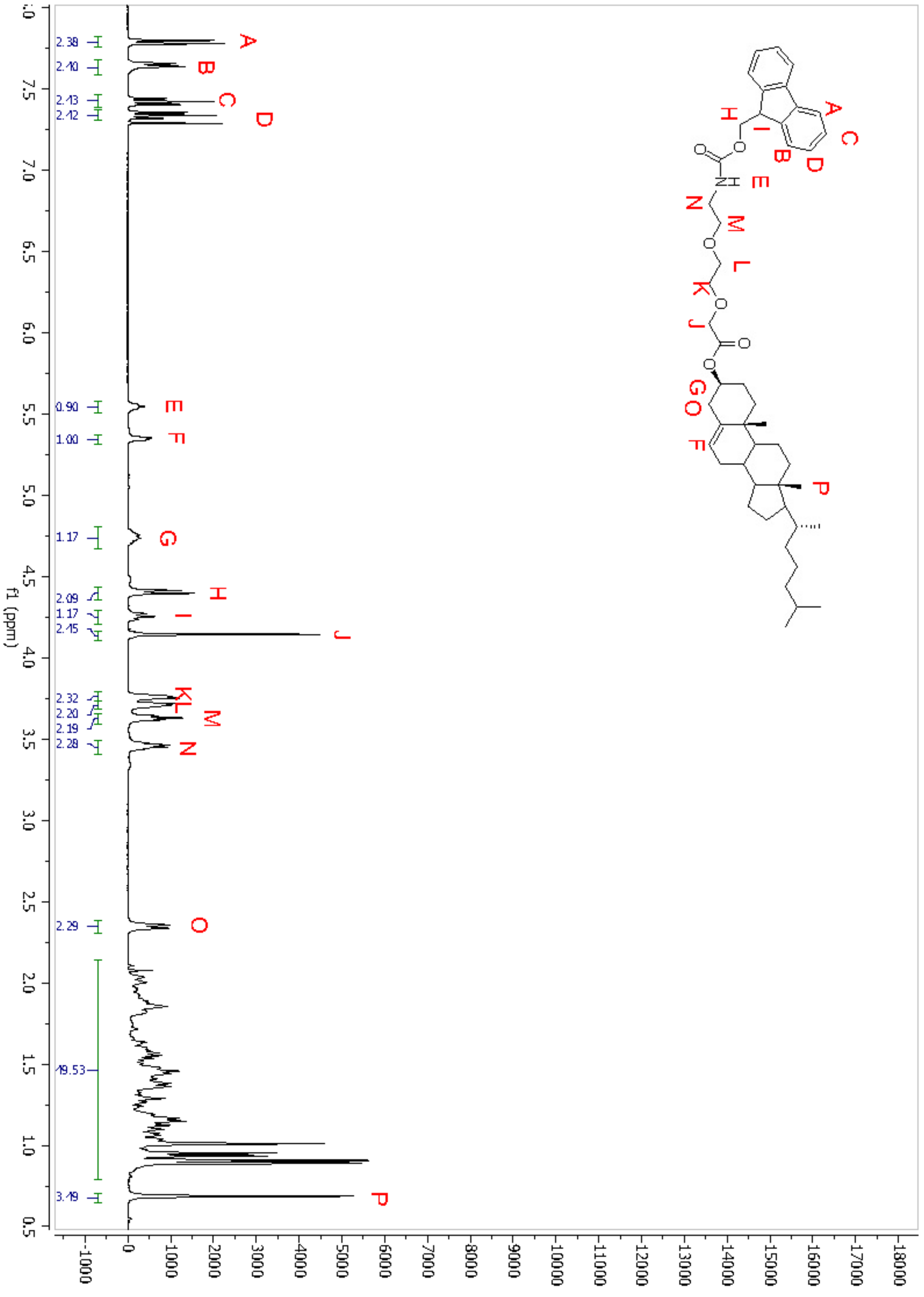

Supplementary Figure 14. ^{1}H-NMR of Cholesteryl-PEG$_2$-NHFmoc

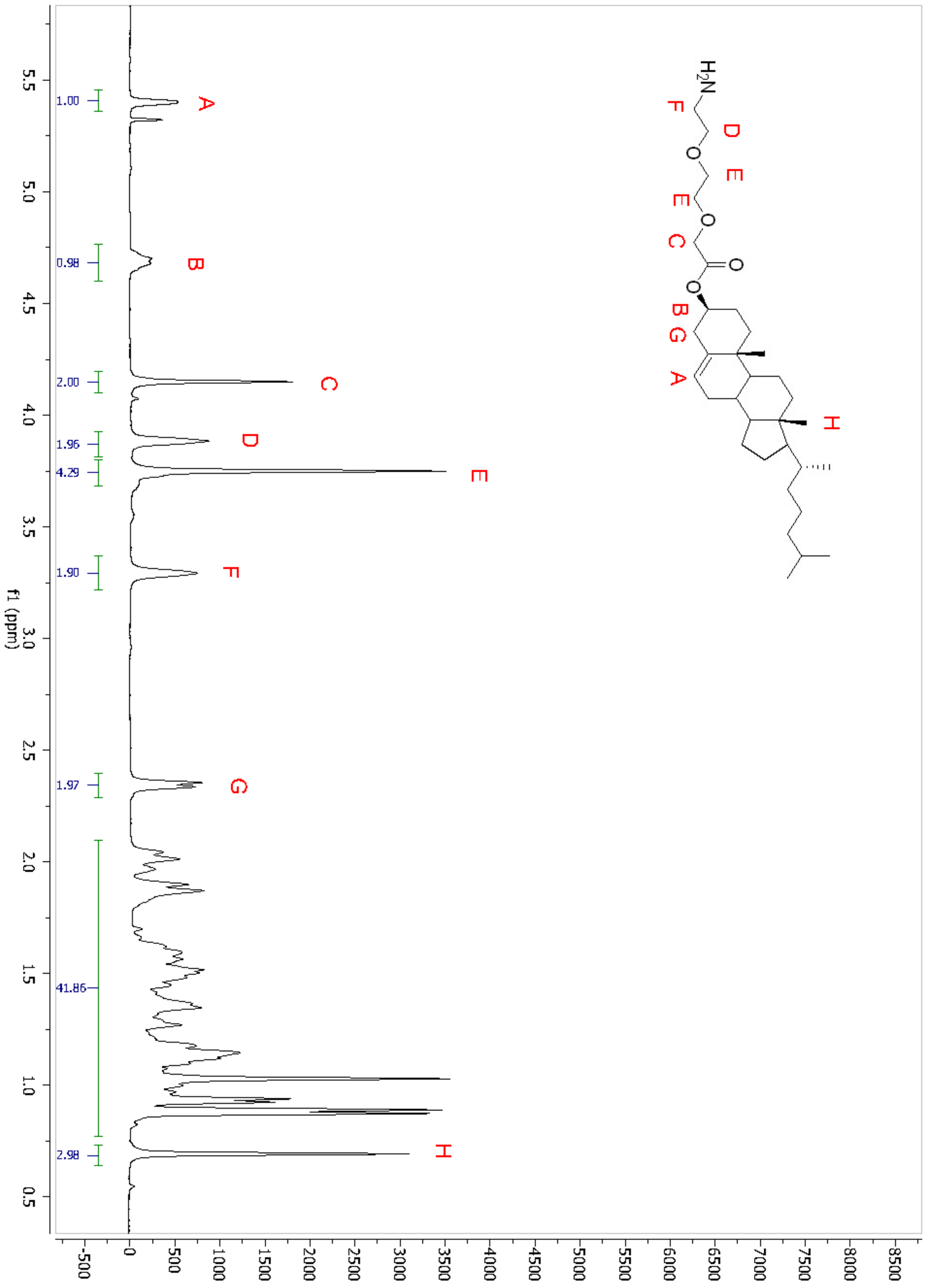

Supplementary Figure 15. ^{1}H-NMR of Cholesteryl-PEG$_2$-NH$_2$, **2**

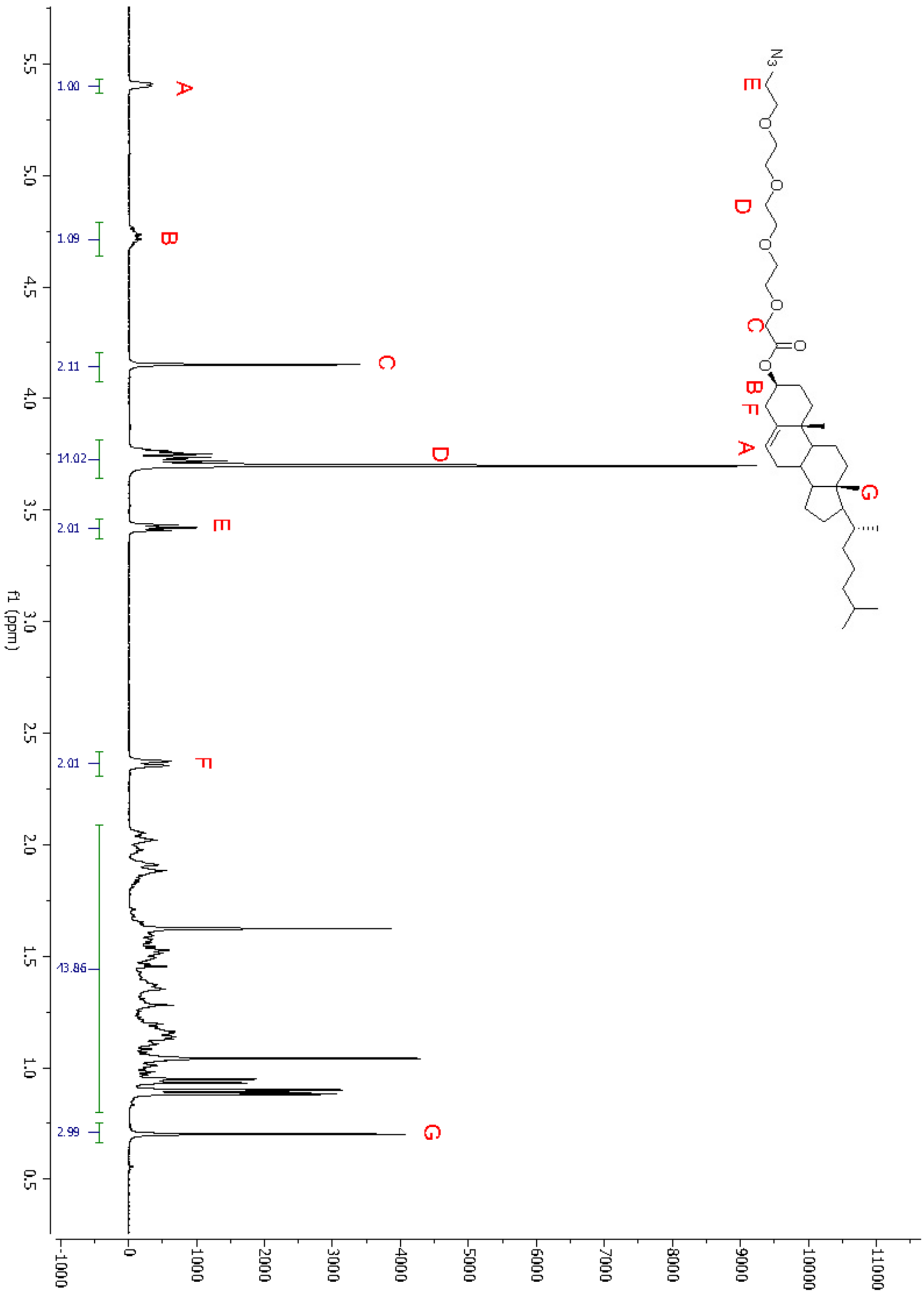

Supplementary Figure 16. ^{1}H-NMR of Cholesteryl-PEG$_4$-N$_3$

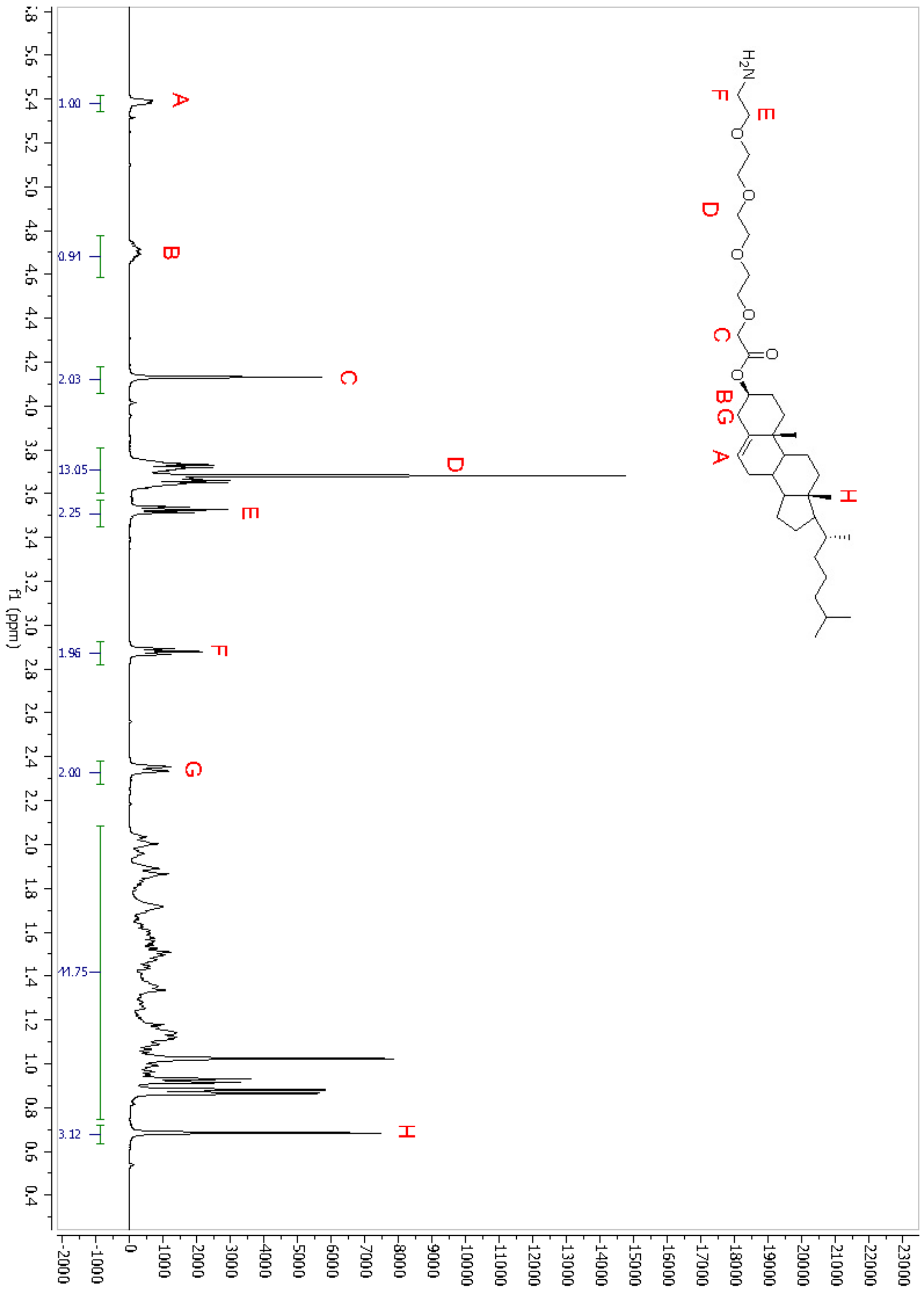

Supplementary Figure 17. ^{1}H-NMR of Cholesteryl-PEG$_4$-NH$_2$, **3**

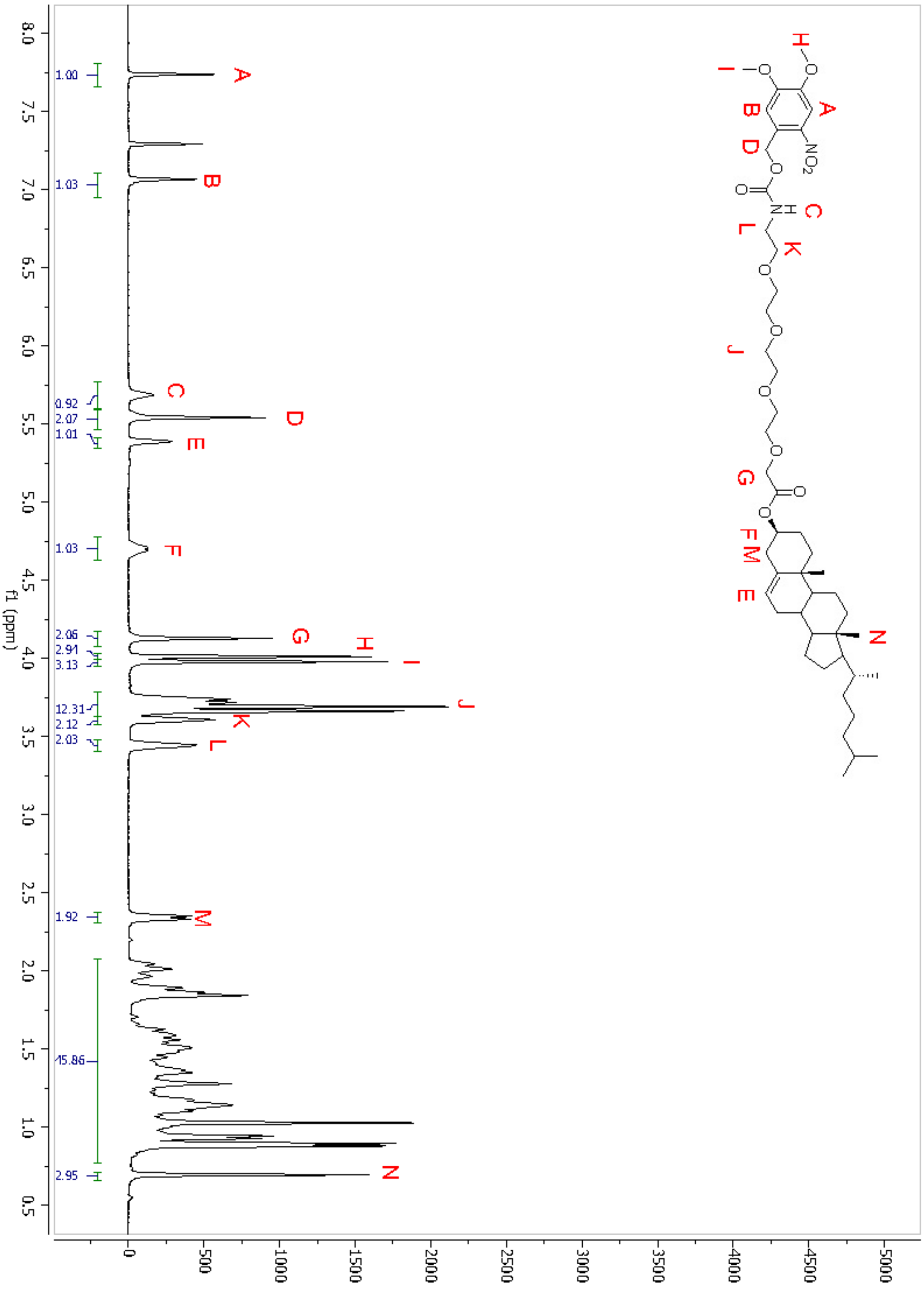

Supplementary Figure 18. ^{1}H-NMR of Cholesteryl-PEG$_4$-NVOC, **4**

Supplementary references

1. Kemmer, G. C. *et al.* Lipid-conjugated fluorescent pH sensors for monitoring pH changes in reconstituted membrane systems. *Analyst* **140**, 6313–6320 (2015).
2. de Belder, A. N. & Wik, K. O. Preparation and properties of fluorescein-labelled hyaluronate. *Carbohydr. Res.* **44**, 251–257 (1975).
3. Aleström, P. *et al.* Zebrafish: Housing and husbandry recommendations. *Lab. Anim.* **0**, 1–12 (2019).
4. Jin, S. W. S.-W., Beis, D., Mitchell, T., Chen, J. N. & Stainier, D. Y. R. Cellular and molecular analyses of vascular tube and lumen formation in zebrafish. *Development* **132**, 5199–5209 (2005).
5. Ellett, F., Pase, L., Hayman, J. W., Andrianopoulos, A. & Lieschke, G. J. mpeg1 promoter transgenes direct macrophage-lineage expression in zebrafish. *Blood* **117**, 49–56 (2011).
6. Weinstein, B. M., Stemple, D. L., Driever, W. & Fishman, M. C. Gridlock, a localized heritable vascular patterning defect in the zebrafish. *Nat. Med.* **1**, 1143–1147 (1995).
7. Schindelin, J. *et al.* Fiji: an open-source platform for biological-image analysis. *Nat. Methods* **9**, 676–82 (2012).
8. Schneider, C. A., Rasband, W. S. & Eliceiri, K. W. NIH Image to ImageJ: 25 years of image analysis. *Nat. Methods* **9**, 671–675 (2012).
9. Campbell, F. *et al.* Directing Nanoparticle Biodistribution through Evasion and Exploitation of Stab2-Dependent Nanoparticle Uptake. *ACS Nano* **12**, 2138–2150 (2018).
10. Van den Broek, B., Oosterkamp, T. H. & van Noort, J. A Multifocal Two-Photon Microscopy Setup for Parallel 3D Tracking of Gold Nanorods. *Biophys. J.* **98**, 178a (2010).
11. Svobodová, H., Nonappa, Lahtinen, M., Wimmer, Z. & Kolehmainen, E. A steroid-based gelator of A(LS)2 type: tuning gel properties by metal coordination. *Soft Matter* **8**, 7840–7847 (2012).
