# Supplementary material for: Light-triggered switching of liposome surface charge directs delivery of membrane impermeable payloads in vivo
Source: Nat Commun. 2020 Jul 20;11:3638. doi: 10.1038/s41467-020-17360-9 (PMC7371701; doi:10.1038/s41467-020-17360-9)
Supplement: Supplementary file 3 — Description of Additional Supplementary Files [file 41467_2020_17360_MOESM3_ESM.docx]

Description of Additional Supplementary Files

**Supplementary Movie 1**. Two-photon live imaging (1 fps) of DOPC:4 liposome distribution before and during UV irradiation. DOPC:4 liposomes contained 1 mol% fluorescent lipid probe, DOPE-LR, for visualization. Plane of view (200 µm x 200 µm) to include both dorsal aorta (DA), posterior cardinal vein (PCV) and a single intersegmental vessel (ISV) connected to, and extending dorsally from, the PCV. (Top) Live imaging for 7 mins (1 fps) before UV irradiation (t = -7.00 → -0.01). UV irradiation initiated at t = 0.00 (95% UV duty cycle). Simultaneous UV and imaging continued for 15 mins (t = 0.01 → 15.00). (Bottom) Control experiment showing real-time (1 fps) DOPC:4 liposome distribution in the absence of UV irradiation (t = -7.00 → 15.00).

**Supplementary Movie 2.** Two-photon live imaging (1 fps) of SR-B filled, DOPC:4 liposome distribution before and during UV irradiation. Plane of view (200 µm x 200 µm) to include dorsal aorta (DA), caudal hematopoietic tissue (CHT) and caudal vein (CV). Imaging for 5 mins (1 fps) before UV irradiation (t = -5.00 → -0.01). UV irradiation initiated at t = 0.00 (95% UV duty cycle). Simultaneous UV and imaging continued for 15 mins (1 fps) (t = 0.01 → 15.00).
