# Supplementary material for: Light-triggered switching of liposome surface charge directs delivery of membrane impermeable payloads in vivo
Source: Nat Commun. 2020 Jul 20;11:3638. doi: 10.1038/s41467-020-17360-9 (PMC7371701; doi:10.1038/s41467-020-17360-9)
Supplement: Supplementary file 6 — Reporting Summary [file 41467_2020_17360_MOESM6_ESM.pdf]

nature research

Corresponding author(s): Frederick Campbell
Alexander Kros

Last updated by author(s): Jun 8, 2020

Reporting Summary

Nature Research wishes to improve the reproducibility of the work that we publish. This form provides structure for consistency and transparency in reporting. For further information on Nature Research policies, see Authors & Referees and the Editorial Policy Checklist .

Statistics

For all statistical analyses, confirm that the following items are present in the figure legend, table legend, main text, or Methods section.

n/a	Confirmed	
☒	☐	The exact sample size (*n*) for each experimental group/condition, given as a discrete number and unit of measurement
☒	☐	A statement on whether measurements were taken from distinct samples or whether the same sample was measured repeatedly
☒	☐	The statistical test(s) used AND whether they are one- or two-sided<br>*Only common tests should be described solely by name; describe more complex techniques in the Methods section.*
☒	☐	A description of all covariates tested
☒	☐	A description of any assumptions or corrections, such as tests of normality and adjustment for multiple comparisons
☒	☐	A full description of the statistical parameters including central tendency (e.g. means) or other basic estimates (e.g. regression coefficient) AND variation (e.g. standard deviation) or associated estimates of uncertainty (e.g. confidence intervals)
☒	☐	For null hypothesis testing, the test statistic (e.g. *F*, *t*, *r*) with confidence intervals, effect sizes, degrees of freedom and *P* value noted<br>*Give P values as exact values whenever suitable.*
☒	☐	For Bayesian analysis, information on the choice of priors and Markov chain Monte Carlo settings
☒	☐	For hierarchical and complex designs, identification of the appropriate level for tests and full reporting of outcomes
☒	☐	Estimates of effect sizes (e.g. Cohen's *d*, Pearson's *r*), indicating how they were calculated

Our web collection on statistics for biologists contains articles on many of the points above.

Software and code

Policy information about availability of computer code

Data collection	Zetasizer Nano ZS software (version 7.13, Malvern Panalytical)<br>TECAN M-1000 i-control microplate reader software (no version information, TECAN)<br>Cary WinUV software (version 3.0, Agilent)<br>For confocal microscopy: Leica Application Suite X (LAS X, version 3.5.5.19976, Leica Microsystems)<br>For 2-photon microscopy: Custom-built LabVIEW software (version 2018 SP2, National Instruments)
Data analysis	Confocal images were analysed using the Fiji distribution of ImageJ, versions 1.51p and 1.52p (Schindelin, J. et al. Fiji: an open-source platform for biological-image analysis Nature Methods 2012, 9, 676-682).<br>Quantification (Figure 4g) was performed using analysis methods previously reported (Campbell, F. et al. Directing Nanoparticle Biodistribution through Evasion and Exploitation of Stab2-Dependent Nanoparticle Uptake. ACS Nano, 2018, 12, 2138-2150)<br>No new custom code was used to analyse the data in this study.

For manuscripts utilizing custom algorithms or software that are central to the research but not yet described in published literature, software must be made available to editors/reviewers. We strongly encourage code deposition in a community repository (e.g. GitHub). See the Nature Research guidelines for submitting code & software for further information.

Data

Policy information about availability of data

All manuscripts must include a data availability statement. This statement should provide the following information, where applicable:
- Accession codes, unique identifiers, or web links for publicly available datasets
- A list of figures that have associated raw data
- A description of any restrictions on data availability

Data supporting the findings of this paper are available from the corresponding authors upon reasonable request. Source data (raw confocal z-stacks and collated data as single Excel sheet) underpinning the data presented in Figure 4g have been deposited within the public image database, fighare.com (DOI: 10.6084/

m9.figshare.12387629).

Field-specific reporting

Please select the one below that is the best fit for your research. If you are not sure, read the appropriate sections before making your selection.

☒ Life sciences ☐ Behavioural & social sciences ☐ Ecological, evolutionary & environmental sciences

For a reference copy of the document with all sections, see nature.com/documents/nr-reporting-summary-flat.pdf

Life sciences study design

All studies must disclose on these points even when the disclosure is negative.

Sample size	No statistical methods were used to calculate sample size. For all experiments in embryonic zebrafish, the sample size was >20. This sample size was determined based on similar reported studies and balances the practicalities involved in maintaining strict confocal imaging timeframes with the need for a large enough population to ensure reproducible results. Where liposome fate was analyzed before and after UV activation, the same injected embryo was imaged before and after UV irradiation.
Data exclusions	With the exception of unsuccessfully injected embryos, no data was excluded from the analysis.
Replication	All experiments were repeated at least twice, with the exception of data presented in Figure 6b,c and Supplementary Figures 1 and 9. All replicate experiments were performed using freshly prepared liposomes. Unless clearly stated in the manuscript text (e.g. varying macrophage uptake prior to UV activation), all replicate experiments were successful and confirmed the presented data.
Randomization	For all experiments in embryonic zebrafish, at least four embryos were randomly selected for imaging from a pool of >20 successfully injected embryos.
Blinding	Blinding was not applied in this proof-of-concept study as all experiments were performed without prior knowledge of the experimental outcome. Key results were observed by more than two different investigators.

Reporting for specific materials, systems and methods

We require information from authors about some types of materials, experimental systems and methods used in many studies. Here, indicate whether each material, system or method listed is relevant to your study. If you are not sure if a list item applies to your research, read the appropriate section before selecting a response.

Materials & experimental systems

n/a	Involved in the study	
☒	☐	Antibodies
☒	☐	Eukaryotic cell lines
☒	☐	Palaeontology
☐	☒	Animals and other organisms
☒	☐	Human research participants
☒	☐	Clinical data

Methods

n/a	Involved in the study	
☒	☐	ChIP-seq
☒	☐	Flow cytometry
☒	☐	MRI-based neuroimaging

Animals and other organisms

Policy information about studies involving animals; ARRIVE guidelines recommended for reporting animal research

Laboratory animals	The following established zebrafish (Danio rerio) transgenic lines were used in this study:<br>Tg(kdrl:eGFP)s843 - (Jin, S. W. S.-W., Beis, D., Mitchell, T., Chen, J. N. & Stainier, D. Y. R. Cellular and molecular analyses of vascular tube and lumen formation in zebrafish. Development, 2005, 132, 5199–5209)<br>Tg(mpeg1:GFP)gl22, Tg(mpeg1:mCherry)gl23 - (Ellett, F., Pase, L., Hayman, J. W., Andrianopoulos, A. & Lieschke, G. J. mpeg1 promoter transgenes direct macrophage-lineage expression in zebrafish. Blood, 2011, 117, 49–56.)<br>All liposomes were injected into zebrafish embryos (both male and female) between 52-56 hours post-fertilisation.
Wild animals	This study does not involve wild animals.
Field-collected samples	This study does not involve samples collected from the field.
Ethics oversight	Adult zebrafish (Danio rerio, strain AB/TL) were maintained and handled according to the guidelines described in (Aleström, P. et al. Zebrafish: Housing and husbandry recommendations. Lab. Anim. (2019)) and in compliance with the directives of the local animal welfare committee of Leiden University. No ethical approval was required for experiments in embryonic zebrafish as all embryos used in this study were < 120 hours post-fertilisation.

Note that full information on the approval of the study protocol must also be provided in the manuscript.
